# Prevalence and Correlates of Suicidal Ideation and Deliberate Self-harm in Canadian Adolescents During the Coronavirus Disease 2019 Pandemic

**DOI:** 10.1177/07067437211036612

**Published:** 2021-08-11

**Authors:** Brianna J. Turner, Christina L. Robillard, Megan E. Ames, Stephanie G. Craig

**Affiliations:** 1 Department of Psychology, 8205 University of Victoria, Victoria, British Columbia; 2 Department of Psychology, 7991 York University, Toronto, Ontario

**Keywords:** suicide, self harm, adolescence, child and adolescent psychiatry

## Abstract

**Objective:**

In light of recent evidence that the coronavirus disease 2019 (COVID-19) pandemic has resulted in marked increases in depression, anxiety, substance use, and other mental health concerns among Canadian adolescents, we investigated the rates of self-harm thoughts and behaviours in this population. Specifically, this study explored: (1) the demographic and geographic distributions of suicidal ideation (SI) and deliberate self-harm (DSH), and (2) the associations of mental health and substance use with SI and DSH.

**Method:**

A total of 809 Canadian adolescents, aged 12–18 years, completed an online survey between June 17, 2020 and July 31, 2020.

**Results:**

44% of adolescents reported experiencing SI since the pandemic began, while 32% reported engaging in DSH. SI and DSH were more common among youth who: identified as transgender, non-binary or gender fluid; who did not reside with both parents; and who reported psychiatric concerns or frequent cannabis use.

**Conclusion:**

Canadian adolescents appear to be experiencing higher rates of self-harm thoughts and behaviours relative to before the COVID-19 pandemic. It is important for adults who are likely to interact with distressed youth to be aware of potential warning signs that a youth is struggling with self-harm, and to refer youth to specialty mental health services where appropriate.

Canadian adolescents reported markedly higher-than-usual rates of anxiety, depression, and substance use during the coronavirus disease 2019 (COVID-19) pandemic.^1^ Rates of recent suicidal ideation (SI) reportedly tripled, from 6%^2^ to 18%.^1^ We aimed to extend knowledge by examining: (1) which adolescents are most vulnerable to SI and (2) whether we observe similar elevations in deliberate self-harm (DSH), defined as intentional self-injury with non-suicidal or suicidal intent. To do so, we assessed demographic and psychiatric correlates of SI and DSH in a convenience sample of Canadian adolescents recruited during the first 4 months of the COVID-19 pandemic.

## Method

Adolescents (*n* = 809; 56% cis-gender female, 74% White, *M*_age_ = 15.67, *SD* *=* 1.37) were recruited via social media advertisements from June 17, 2020 to July 31, 2020. Most youth (85%, *n* = 688) who started the online survey completed it, and scale-level missing data were minimal (<1% for all variables except ethnicity [7%] and substance use frequencies [15%]). Recent (past 4-month) conduct problems, oppositionality, attention problems/hyperactivity, depression, anxiety, separation fears, and social anxiety were assessed with the 52-item Ontario Child Health Study Scales^3^ (subscale αs = .70–.87). Recent (past 90-day) use of alcohol, cannabis, and illicit drugs (e.g. ecstasy/MDMA, opiates) was measured using the 14-item Drug History Questionnaire.^4^ Participants reported whether, during the past 4 months, “*I think about killing myself*” (SI) or “*I deliberately try to hurt or kill myself*” (DSH) were “never, or not true” (0), “sometimes, or somewhat true” (1), or “often, or very true” (2). Analyses dichotomized SI and DSH as present (1 or 2) or absent (0). Full study details are available at https://psyarxiv.com/kprd9/.

## Results

Our sample closely matched known demographics of Canadian youth, with similar proportions of youth identifying as visible minorities (26% vs 27%^2^), living in two-parent households (70% in both^2^), reporting any history of alcohol use (42% vs 44%^2^) and accessing mental health supports (17% in both^2^). Higher rates of psychiatric concerns in this sample (e.g. major depression: 50%) versus representative pre-pandemic surveys (11%^2^) may be attributable to use of a screening measure^3^ here versus prior diagnoses.^2^

 Table 1 summarizes results. One-quarter of adolescents (26%, *n* *=* 212) reported both SI and DSH, one in six (17%, *n* = 139) reported SI only, and one in 20 (5%, *n* = 43) reported DSH only. There were no regional or racial/ethnic differences in SI or DSH. Transgender/non-binary youth reported more SI and DSH than cis-gender youth, and cis-gender females reported more DSH than cis-gender males. Youth who lived with both parents reported less SI and DSH than youth who lived with a single parent, divided time between multiple households, or did not live with parents.

**Table 1.** Rates of SI and DSH by Demographic Group, and Predicted by Psychiatric Concerns or Substance Use Frequency*.*

	Endorsing SI (*n* = 352)			Endorsing DSH (*n* = 256)		
	% (*n*)	*χ*^2^ (df)	*P*	% (*n*)	*χ*^2^ (df)	*P*
BC^a^	47 (55)	5.76 (5)	0.33	33 (29)	4.83 (5)	0.44
Alberta^a^	45 (72)			35 (56)		
Prairies^a^	46 (41)			30 (27)		
Ontario^a^	44 (119)			31 (85)		
Québec^a^	30 (20)			21 (14)		
Atlantic^a^	43 (42)			34 (33)		
White^b^	42 (252)	1.63 (1)	0.20	47 (99)	1.19 (1)	0.29
Non-White^b^	31 (183)			35 (72)		
Boys	38 (120)	A^c^: 3.30 (1) B^c^: 10.74 (1)	A^c^: *P* = 0.07 B^c^: *P* = 0.001	24 (76)	A^c^: 7.25 (1) B^c^: 26.74 (1)	A^c^: *P* = 0.007 B^c^: *P* *<* 0.001
Girls	45 (204)			33 (152)		
TNBI	68 (28)			68 (28)		
Living with two parents	40 (220)	8.82 (1)	0.003	28 (151)	13.76 (1)	<0.001
Not living with two parents	51 (132)			41 (104)		
	Logistic regression predicting SI			Logistic regression predicting DSH		
*Psychiatric concerns*	OR	95% CI of OR	*P*	OR	95% CI of OR	*P*
Conduct	1.27	1.14, 142	<0.001	1.16	1.05, 1.28	0.003
Oppositional	0.97	0.89, 1.06	0.514	1.01	0.92, 1.10	0.918
ADHD	0.96	0.90, 1.03	0.272	0.98	0.92, 1.05	0.634
Anxiety	1.19	1.10, 1.30	<0.001	1.10	1.01, 1.20	0.024
Depression^d^	1.34	1.24, 1.44	<0.001	1.28	1.18, 1.38	<0.001
Sep. fears	0.98	0.92, 1.04	0.493	0.98	0.93, 1.04	0.554
Social anxiety	1.00	0.92, 1.08	0.998	1.12	1.03, 1.21	0.008
Any elevation (≥1) vs 0^e^	6.36	4.56, 8.88	<0.001	8.97	5.45, 14.74	<0.001
	Logistic regression predicting SI			Logistic regression predicting DSH		
*Frequency of substance use*	OR	95% CI of OR	*P*	OR	95% CI of OR	*P*
Alcohol	1.05	0.91, 1.21	0.485	1.12	0.97, 1.29	0.129
Cannabis	1.33	1.16, 1.53	<0.001	1.16	1.03, 1.31	0.016
MDMA	0.94	0.27, 3.25	0.917	1.28	0.45, 3.64	0.640
Opiates	1.99	0.54, 7.27	0.301	0.92	0.37, 2.25	0.847
Prescription	1.04	0.82, 1.31	0.755	1.07	0.85, 1.35	0.530
Nicotine	1.05	0.94, 1.17	0.353	1.03	0.93, 1.14	0.622
Other	1.25	0.83, 1.86	0.286	1.11	0.79, 1.57	0.556
Any versus no use	1.47	1.26, 1.71	<0.001	1.41	1.18, 1.68	<0.001
Any illicit use versus ETOH only	1.50	1.15, 1.96	0.002	1.27	0.96, 1.69	0.088

*Notes:* All logistic regression models controlled for gender (coded as 0 = male, 1 = female or TNBI) and living situation of youth (coded as 0 = residing with both parents, 1 = residing with a single caregiver, multiple households, or with someone other than a biological parent).

TNBI: transgender, non-binary or gender fluid; Sep. fears: separation fears; ETOH: alcohol; SI: suicidal ideation; DSH: deliberate self-harm.

^a^ Rates of SI and DSH among participants living in the Territories are not reported due to the small number of respondents (*n* < 5).

^b^ We repeated racial/ethnic contrast excluding respondents who identified as Indigenous, given potentially higher rates of SI and DSH in Indigenous Canadian youth relative to other racial and ethnic minorities and the small number of Indigenous respondents in this sample (*n* = 54). Results were comparable, indicating that non-Indigenous minority youth had similar rates of SI (44%) and DSH (30%) as non-Indigenous White youth (42% and 31%, respectively; *χ*^2^[1] = 0.13, *P* = 0.72 and *χ*^2^[1] = 0.06, *P* = 0.81).

^c^ Contrast A compares cis-gender girls and cis-gender boys. Contrast B compares TNBI youth and cis-gender youth.

^d^ Depression is scored based on seven of the nine items of the Ontario Child Health Study Scales, excluding the two items pertaining to suicidal ideation and deliberate self-harm.

^e^ We used weighted population norms to identify a clinical cutoff score that most closely matched the population prevalence of the corresponding disorder.

Psychiatric concerns accounted for 43% and 33% of the variance in SI and DSH, respectively. Depression, anxiety, and conduct problems were uniquely associated with SI and DSH, and social anxiety was uniquely associated with DSH. Youth with at least one clinically significant psychiatric concern had 6–9 times greater odds of SI and DSH, versus those who scored below all clinical cutoffs.

Frequency of substance use accounted for 9% and 4% of the variance in SI and DSH, respectively. Only cannabis use was uniquely associated with SI and DSH, controlling for other substances. Youth with *any* recent alcohol, cannabis, or illicit drug use had 40% greater odds of SI and DSH than youth who reported no alcohol or drug use. Compared to youth who used alcohol *only*, youth who used cannabis or illicit drugs reported 50% higher odds of SI.

## Discussion

Identifying demographic features associated with self-harm can inform targeted assessment and intervention, while identifying modifiable risk factors such as psychiatric concerns can inform what types of supports may be beneficial. Our results identified gender minority youth and youth in single-parent homes as being at especially high risk of SI and DSH, suggesting a need for additional screening of these potentially vulnerable youth. Moreover, we identified depression, anxiety, conduct problems, and frequent cannabis use as associated with risk of SI and DSH, suggesting the potential value of coupling targeted suicide prevention (e.g. safety planning, lethal means restriction) with interventions for internalizing and externalizing concerns.

Several limitations merit consideration. Firstly, convenience samples remain susceptible to self-selection biases and caution must be applied to any survey with unobserved variables influencing participation. Secondly, this study's cross-sectional design prevented comparisons across phases of the pandemic. Thirdly, we did not differentiate DSH *with* versus *without* suicidal intent, and so were unable to investigate rates of non-suicidal self-injury. Finally, we note that available data suggest suicide deaths remained steady or slightly declined in the early stages of the pandemic,^5^ and trends for suicidal thoughts, non-fatal self-harm, and fatal self-harm often diverge. Ongoing monitoring of population-level indicators across this spectrum of risk is warranted as the pandemic and its aftermath unfold. This study nonetheless augments literature on the well-being of Canadian adolescents during the COVID-19 pandemic and bolsters calls to enhance support for youth in crisis.
